# TKIs combined with chemotherapy followed by allo-HSCT in Philadelphia chromosome-positive myelodysplastic syndrome: A case report and literature review

**DOI:** 10.1097/MD.0000000000031874

**Published:** 2022-11-18

**Authors:** Shasha Qi, Feiqing Wang, Yang Liu, Jiangyuan Zhao, Yan Wang, Songsong Huang, Wenxiu Yang, Yanling Li, Yong Shen, Chike Zhang, Jianing Zhao, Xu Yang, Rui Gao, Ying Chen, Peng Zhao, Fengqi Zhang, Yi Huang, Mei Zhao, Ping Wang, Yan Zhang, Hanbo Dou, Jishi Wang, Yanju Li

**Affiliations:** a Guizhou Medical University, Guiyang, China; b Clinical Research Center, The First Affiliated Hospital of Guizhou University of Traditional Chinese Medicine, Guiyang, China; c Department of Hematology, Affiliated Hospital of Guizhou Medical University, Guiyang, China.

**Keywords:** allogeneic hematopoietic stem cell transplantation, myelodysplastic syndrome, Philadelphia chromosome, tyrosine kinase inhibitors

## Abstract

Philadelphia chromosome (Ph) positive myelodysplastic syndrome (MDS) is a very rare disease. At present, the specific role of Ph in MDS is not clear, but such patients seem to have a poor prognosis, so the disease deserves attention. Here, we describe the history of a woman with Ph-positive MDS and perform a systematic review of related literature.

**Patient concerns and diagnosis:**

We report a 38-year-old woman with Ph-positive MDS.

**Interventions and outcomes:**

She received chemotherapy with decitabine, cytarabine, aclarubicin, and granulocyte colony-stimulating factor (DCAG) combined with imatinib mesylate and achieved a bone marrow remission. She then underwent an allogeneic hematopoietic stem cell transplant. The condition is good and no recurrence of the disease has been observed.

**Conclusion:**

Ph-positive MDS is a very rare disease. Ph may aid in the malignant progression of MDS leaving such patients with a very poor prognosis. Tyrosine kinase inhibitors (TKIs) plus chemotherapy followed by allogeneic hematopoietic stem cell transplantation has provided these patients with satisfactory outcomes.

## 1. Introduction

Myelodysplastic syndrome (MDS) is an acquired clonal stem cell disorder that can easily develop into acute myeloid leukemia (AML). It is often associated with various chromosomal abnormalities.^[1]^ In statistical models of individual prognoses, such as the International Prognostic Scoring System, chromosomal karyotype has been demonstrated to be one of the most significant prognostic parameters of MDS.^[2]^ Philadelphia chromosome (Ph) is produced by a reciprocal translocation between the long arms of chromosome 9 and chromosome 22, that is, t (9; 22) (q34; q11).^[3]^ This chromosome is mostly found in chronic myelogenous leukemia (CML), but it also appears in acute lymphoblastic leukemia. On the contrary, the Ph is rare in AML and MDS, with only a few cases reported in the latter. Because this is such a rare disease, it is unclear whether the Ph has any clinical value in MDS or what specific role it plays in the pathogenesis, prognosis, and progression. We report here one primary Ph-positive case of MDS, along with a brief literature review in order to raise awareness of Ph-positive MDS.

## 2. Case report

**Figure 1.**
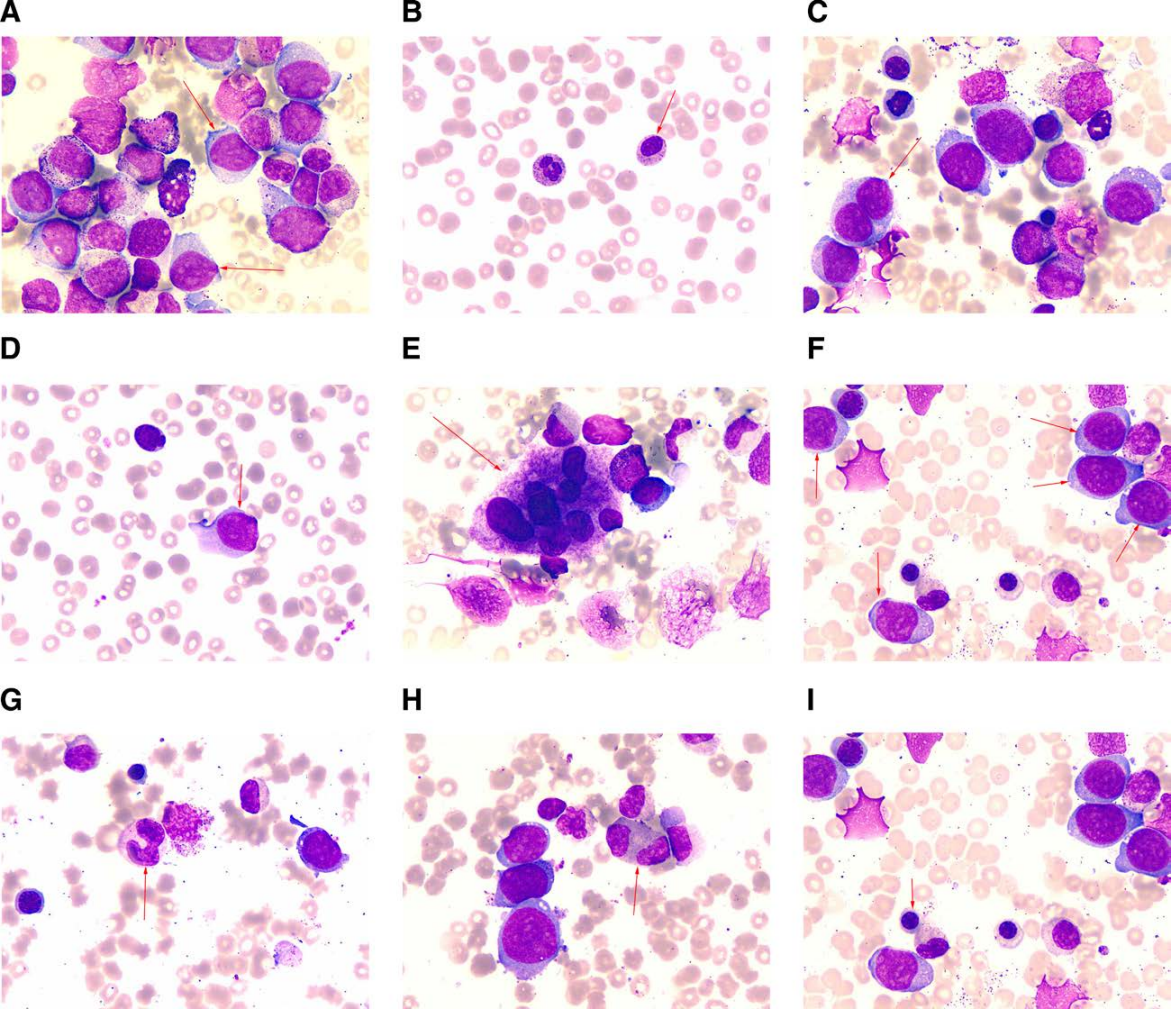
The patient’s bone marrow and peripheral blood smear results. (A) = The primitive cell in the bone marrow, (B) peripheral blood smear shows naive granulocytes, (C) double nuclei of granulocytes seen in a bone marrow smear, (D) primitive cells in a peripheral blood smear, (E) Polyprotogenic giant cells were seen in a bone marrow smear, (F) primitive granulocytes in the bone marrow, (G) giant rod-shaped granulocytes in the bone marrow, (H) a granulocyte with two nuclei in the bone marrow, and (I) mild imbalance of erythrocyte development in the bone marrow.

**Figure 2.**
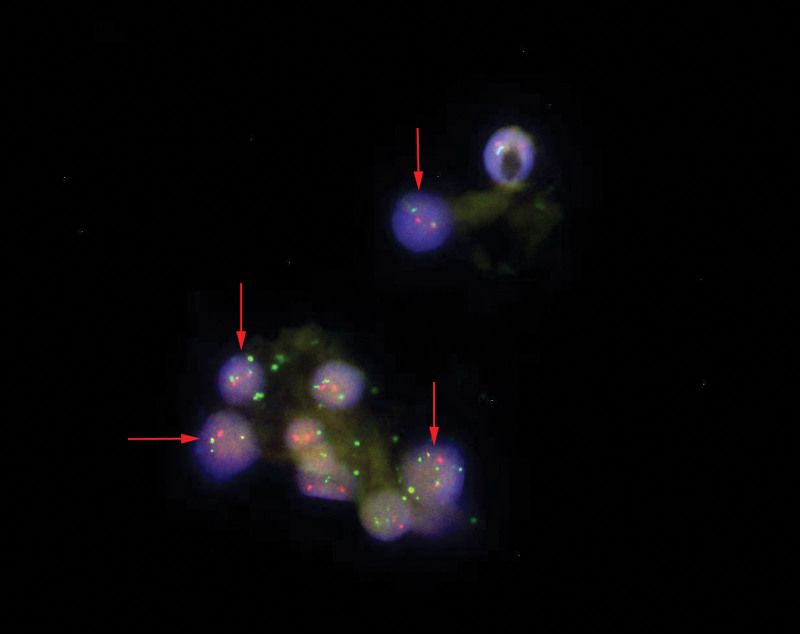
The patient t (9; 22) chromosome karyotype analysis result.

We present the case of a 38-year-old female patient who was in her usual state of health until February 3, 2021, when she developed a fever of no known cause. She reported to the Affiliated Hospital of Guizhou Medical University and was admitted. Related auxiliary examination after hospitalization: white blood cell counts 12.6 × 10^9^/L, neutrophil absolute value 9.79 × 10^9^/L, red blood cell (RBC) counts 2.2 × 10^12^/L, hemoglobin 66.00g/L, mean RBC volume 95.00 fL, platelet count 57.00 × 10^9^/L. A bone marrow examination showed hyperactive myelodysplasia with 17% primary cells. Granulocytes were hyperactive with toxic changes, and a few granulocytes reduced cell particles. Large rod-shaped nuclear and dinuclear granulocytes were occasionally seen. Erythroid hyperplasia was present, and the cytoplasmic and nuclear development of some young erythrocytes was slightly unbalanced. The mature erythrocytes were uneven in size and polychromatic erythrocytes were seen. Lymphocytes accounted for 10%. There were 107 megakaryocytes, including primitive megakaryocytes (1%), young megakaryocytes (1%), granulated megakaryocytes (89%), and platelet-producing megakaryocytes (9%). Some megakaryocyte nuclei had an excess of lobules, round megakaryocytes were occasionally seen. Platelets were observed scattered in clusters (Fig. 1). Peripheral blood examination showed that the distribution of white blood cells was generally normal, with protocells (6%), young red blood cells, and granulocyte. Dinuclear granulocytes and granulocyte nucleus lobulation failure were observed on rare occasions. The karyotype was determined to be 46, XX, t(9;22) (q34;q11)[1]/46, XX[19] (Fig. 2). She was definitively diagnosed with Ph chromosome-positive refractory anemia with excess blasts (RAEB)-2. Decitabine, cytarabine, aclarubicin, and granulocyte colony-stimulating (DCAG) (decitabine 21 mg/d was given intravenously from day 1 to day 5, cytarabine 0.014 g/12 h was given subcutaneously from day 3 to day 9, aclarubicin 10 mg/d was given intravenously from day 3 to day 9, and human granulocyte colony-stimulating factor 300 μg/d was given subcutaneously from day 3 to day 9) chemotherapy combined with oral imatinib mesylate 400 mg/d targeted therapy was administered. She achieved morphological remission of her bone marrow following a course of chemotherapy. The BCR/ABL gene copy number was zero on April 30, 2021. After 4 courses of DCAG combined with imatinib mesylate, the copy number of the BCR/ABL gene was zero in multiple subsequent examinations. The patient was then discharged after hematopoietic reconstruction in August 2021 after receiving full identical allogeneic hematopoietic stem cell transplantation. She returned for reevaluation on November 3, 2021, and we found that her BCR/ABL copy numbers were zero. Currently, the patient has no signs of disease recurrence and is still being followed up.

## 3. Discussion

MDS refers to a group of bone marrow diseases characterized by high heterogeneity in morphological manifestations, clinical processes, and cytogenetic characteristics, with cytogenetic heterogeneity being the abnormal karyotype in approximately 50% of patients with primary MDS and 80% in patients with secondary MDS.^[1]^ Meanwhile, the common chromosome karyotype abnormality is one of the most important prognostic parameters of MDS according to the International Prognostic Scoring System score (e.g., -5/del (5q), -7/del (7q), +8, del (20q), i(17q)/t(17p), and -y,) (1). Despite all these, about 14% of cytogenetic abnormalities in MDS are of unknown significance.^[1]^

Ph is a marker of CML, which is very rare in patients with MDS.^[3]^ Only a handful of cases have been reported, hence, information on Ph-positive MDS is scarce. What role does the Ph play in MDS? By what mechanism does it work? How should it be treated? No literature provides convincing results. Here, we review and summarize previous case reports and make a preliminary discussion in addition to the case reported in this paper.

To understand the specific role of the Ph in MDS, we searched major medical databases for all case reports of newly diagnosed Ph-positive MDS and found 13 patients^[4–13]^ (Table 1). We analyzed the clinical data of these 13 patients and found that 12 patients died or progressed to leukemia within a year, and 1 patient experienced a 15% increase in bone marrow blasts from 2% within 8 months. As a result, we suspect that the Ph accelerates the malignant progression of MDS.

**Table 1:** Data on newly diagnosed Ph-positive patients with MDS.

Author	Publication date	Age, years	Gender	Diagnosis	Chromosomal karyotype	Treatment options	Patient outcomes
Khaliqur et al^[4]^	2017	55	Female	RAEB-1	45, XX, −4, t (9, 22) (q34; q11.2)	She was initially given demethylation and then switched to supportive therapy.	She died one month after her diagnosis.
Khaliqur et al^[4]^	2017	31	Male	RAEB-2	44-45, XY, der(4), der(7),-7,-8,t(9, 22)(q34; q11.2), +22,-15	No medical treatment was given.	He died seven days after diagnosis.
Yi-Kong et al^[5]^	2003	59	Male	RAEB-t	46, XY, t(9;22)(q34;q11)	After imatinib mesylate 400 mg/d combined with induction chemotherapy achieved complete response, allogeneic hematopoietic stem cell transplantation was performed.	He developed AML two months after his diagnosis of MDS and achieved complete remission after receiving imatinib mesylate in combination with induction chemotherapy. He then underwent allogeneic hematopoietic stem cell transplantation and survived for a long time with residual leukemia.
Yi-Kong et al^[5]^	2003	66	Female	RAEB	47, XX, +8, t(9;22;16)(q34;q11.2;q23)/ 46,XX, der (12) t (12; 17) (p11.2;q11.2)	Macfarlane oral	She died 10 months after her diagnosis
Darko^[6]^	2017	68	Female	RARS	46, XX, t (9; 22)	Blood transfusion	She died three weeks after diagnosis.
Gregor et al^[7]^	1992	73	Male	MDS	46, XY, t(4;6)(p15;p12),t(9;22)(q34;q11)[6	Unclear	He died 10 months after diagnosis.
Drummond et al^[8]^	2002	67	Female	RAEB-2	46, XY, t(9;22)(q34;q11)	After receiving a combination of daunorubicin and cytosine arabinoside, interferon was added due to a poor response. After administering imatinib 400 mg qd for three months, the Ph became negative and the bone marrow’s primitive cells disappeared.	The patient was diagnosed with MDS and rapidly progressed to AML. No disease recurrence was observed during follow-up after treatment.
Lesesve et al^[9]^	1996	66	Male	RAEB-t	46, XY/45, X, -Y]/ 50, XY, +Y, -3, del(5)(q13q34), +8, +13, +14, add (18)(p11),+22,+min/idem,t(9;22)(q34;q11)	Cytosine arabinoside combined with mitoxantrone and teniposide.	He died three months after his diagnosis.
Lesesve et al^[9]^	1996	64	Male	RAEB-t	46-47, XY, 8,t(9;22)(q34;q11)	Supportive treatment combined with cytarabine and teniposide.	He developed leukemia nine months after his diagnosis of MDS and died 16 days later.
Aristides et al^[10]^	2017	74	Male	RCMD	46, XY, t (9:22)	Dasatinib 20mg	His Ph turned negative 4 months after treatment, but he died of severe infection 7 months later.
Keisuke et al^[11]^	1988	62	Male	RAEB	43,XY,5,7,+8,11,12,13,22,del(11)(q11),+der(11)t(11;22)(q11,-q23;q11,), +del(22)(q11)	No medical treatment was given.	The patient was diagnosed with MDS which turned into leukemia after 20 days. There was no follow-up data.
Carlos et al^[12]^	1999	30	Female	RAEB-2	46xx, t(9;22)(q34;q11.2)	Imatinib 400 mg qd	Death occurred shortly after diagnosis.
Rashmi et al^[13]^	2021	67	Female	MDS with del(5q-)	Ph,5q-	Hydroxyurea	Her disease progressed to RAEB 8 months after she was diagnosed with 5q-syndrome and eventually transformed into leukemia.

MDS = myelodysplastic syndromes, RAEB = refractory anemia with excess blasts, RAEB-1 = refractory anemia with excess blasts-, RAEB-2 = refractory anemia with excess blasts-2, RARS = refractory anemia with ring sideroblasts, RCMD = refractory anemia with multilineage dysplasia.

To further confirm this hypothesis, we searched all previous patients with MDS who initially lacked the Ph but later acquired it. Data on a total of 9 patients were retrieved^[5,7,14–20]^ (Table 2). We analyzed the clinical data of these patients and found that 5 patients did not have the Ph, but acquired it during the progression of MDS to leukemia, 2 patients were initially diagnosed with non-RAEB-t MDS without the Ph, but later developed RAEB-t with the acquisition of the Ph. The vast majority patients of these patients died within a year of disease progression. In the remaining 2 patients, one case in the stage of MDS and converted to leukemia in the early stages of the Ph was not found, then give the patient strong induction has failed to achieve complete remission but can maintain the stability of the disease. However, 1 year later, when the Ph was also discovered, the patient’s disease was progressing with significantly more peripheral blood primitive cells than before. The patient died within 3 months of disease progression even after repeated chemotherapy. Another patient diagnosed with MDS developed acute red leukemia 1 month later and achieved complete remission with CAG treatment, but his leukemia recurred 3 months later, and he went into a second remission with a second CAG treatment and did not relapse for a year. Until then, no Ph had been found. A year later, however, his leukemia returned with the appearance of the Ph. Even when CAG was given again, complete remission was not achieved again, and she died soon after. These results are consistent with our hypothesis that the Ph accelerates the malignant progression of MDS.

**Table 2:** Data on Ph-negative patients with MDS who later acquired the Ph Chromosome.

Author	Publication date	Age, years	Gender	Diagnosis	Chromosomal karyotype	Treatment options	Patient outcomes
Yi-Kong et al^[5]^	2003	71	Male	RAEB	normal→46, XY, t(9;22)(q34; q11)	Hydroxyurea	RAEB was transformed into RAEB-t after the appearance of Ph chromosomes and died 4 months later
Gregor et al^[7]^	1982	63	Male	MDS	normal→46, XY, t(9;22)(q34;q11)	Chemotherapy	He was diagnosed with MDS. After 18 months, the disease progressed from MDS to RAEB-t with the appearance of Ph chromosomes. He died after three months of progression.
Mori et al^[14]^	1993	78	Female	RAEB	Her chromosome karyotype was not reported at the time of initial diagnosis, but the Ph chromosome was detected three months after diagnosis.	Ubenimex and blood transfusion	The disease progressed to AML three months after the diagnosis of MDS, and death occurred two months after the progression.
Masahiro et al^[15]^	2003	73	Male	RAEB	His karyotype was normal when he was initially diagnosed with MDS, and Ph chromosomes were detected 30 months after diagnosis.	Treatment at the MDS stage was unclear, but he was given imatinib mesylate after the disease progressed to AML.	The disease did not progress for 30 months after the initial diagnosis of MDS, but with the presence of Ph chromosomes, his disease progressed to AML. He began treatment with imatinib mesylate and achieved complete remission, but relapsed three months later and died about a year later.
Makotokatsuno et al^[16]^	1994	39	Male	RAEB	46,XY,t(3;3),(y21;q26)→46,XY,t(3:3)(q21;q26)→46,XY,del(l)(p22).t(3;3)(q21:y26)→ 46,XY,t(3;3)(q21:q26),t(9;22)(q34:q11)	He received supportive therapy in the MDS stage and induced chemotherapy in the leukemia stage.	He was diagnosed with MDS and developed leukemia three months later. Within a year after he developed leukemia, the disease did not progress, and he did not have a Ph chromosome at this time. A year later, and with the Ph chromosome, his disease progressed rapidly, and he died after three months of progression.
Depei et al^[17]^	2011	20	Male	RAEB-2	normal→46, XY, t(9,22)	The patient did not receive treatment at the MDS stage. However, at the leukemia stage, he received CAG and HA consolidation chemotherapy.	He was diagnosed with MDS, which progressed to AML a month later. He had a relapse two months after receiving the CAG treatment. He then received a second course of CAG after which he went into complete remission. His disease recurred a year after he received HA consolidated intensive chemotherapy, and Ph chromosomes were detected. His disease progressed rapidly even with CAG, but he died shortly afterward.
Akiko et al^[18]^	2012	76	Male	MDS	46,XY, del(5q), add(16)(q13)→46, XY,del(5q),add(16,(q13)/46,idem,t(9;22)(q34;q11.2)/47,idem,add(1),(q32),+8,t(9;22)	Treatment was not given in the MDS stage and nilotinib was given in the leukemia stage	He was diagnosed with MDS without Ph chromosomes. His disease progressed to AML with the presence of Ph chromosomes after 18 months. He was treated with nilotinib for one month and achieved blood remission, but developed drug resistance after six months
Nicole et al^[19]^	1988	57	Female	RAEB	Ph chromosomes were absent in the MDS stage and detected in the advanced leukemia stage.	Uncleara	The Ph chromosome did not appear when she was diagnosed with MDS, but Ph chromosomes were detected after the disease progressed to leukemia. He died 18 months after the disease progressed to leukemia.
Yajun et al^[20]^	2018	55	Female	RT	46, XX→46,XX, t(9;22)(q34;q11.2)/49, idem, +8, +9, +19	He was treated with retinoic acid, prednisone, thalidomide in the MDS stage and imatinib mesylate plus HA in the AML stage.	He was diagnosed with MDS without Ph chromosomes. After 21 months, with the appearance of Ph chromosomes, he progressed AML and died two months later.

MDS = myelodysplastic syndromes, RAEB = refractory anemia with excess blasts, RAEB-2 = refractory anemia with excess blasts-2, RT = myelodysplastic syndromes-refractory thrombocytopenia.

The case reported in this paper is a 38-year-old female patient. Bone marrow examination showed hyperactive myelodysplasia with 17% primary cells. The patient was initially healthy but developed MDS-RAEB-2 in just a few months w indicating that the patient’s condition may be progressing to leukemia or is at a high risk of progressing to leukemia in a short period.

With the in-depth study of the BCR-ABL fusion gene, targeted tyrosine kinase inhibitors (TKIs) were used in the treatment of CML and achieved remarkable efficacy. However, there is little research on Ph-positive MDS, and it is unclear whether TKIs can achieve similar results as in CML in Ph-positive MDS. It is well known that the morphological manifestations, clinical process, and cytogenetic characteristics of MDS are different from those of CML. If, as we suspect, Ph merely accelerate the malignant progression of MDS rather than being the cause of the disease, TKIs monotherapy may not be able to achieve similar efficacy as in CML. At the same time, because Ph accelerates the malignant progression of MDS, treating MDS without considering Ph may not achieve an optimum effect.

To test our hypothesis and preliminarily explore the standard treatment regimen for Ph-positive MDS, we analyzed Ph-positive MDS, Ph-positive leukemia from MDS progression reported in previous works of literature, and the patient reported in this article.

In 14 newly diagnosed Ph-positive MDS patients, Of the 6 patients who received supportive chemotherapy, 5 died within 1 year of diagnosis, 1 surviving patient had no follow-up data after 8 months. Of the two patients who received TKIs monotherapy, 1 died within 1 year of diagnosis, and the other patient’s Ph disappeared 4 months after treatment but died of severe pneumonia 7 months later. Of the 3 patients who received chemotherapy combined with TKIs, bone marrow morphological response was achieved in 1 case, complete response was achieved in 1 case, and the molecular response was achieved in 1 case. Two of the three patients underwent subsequent allogeneic hematopoietic stem cell transplantation. During follow-up, all 3 patients were in good condition. The remaining 2 patients with unclear treatment or no treatment died within a year.

Nine patients were initially diagnosed with MDS without Ph but later acquired it. Five patients of these patients who received chemotherapy died within a year after the Ph appeared. Two patients these patients who received TKIs monotherapy achieved a blood response and complete response, respectively, within a short period, but all experienced disease recurrence within 1 year. One patient who received a TKIs combined with chemotherapy failed to respond after treatment and gave up treatment. One patient whose treatment was unclear died one year after acquiring the Ph chromosome.

According to these results, the vast majority of patients treated with chemotherapy alone die within a year of diagnosis or acquisition of Ph. Although TKIs monotherapy can achieve some short-term efficacy, patients are prone to relapse. Of the 4 patients treated with TKIs in combination with chemotherapy, 3 achieved good results and 1 had no response. However, 2 of the 3 patients with good outcomes later underwent allogeneic hematopoietic stem cell transplantation, so we could not evaluate the long-term efficacy of TKIs in combination with chemotherapy. Although the long-term efficacy of TKIs combined with chemotherapy cannot be evaluated, allogeneic hematopoietic stem cell transplantation after TKIs combined with chemotherapy did achieve a satisfactory result in these patients, and perhaps this regimen can fundamentally treat such highly malignant or highly advanced Ph-positive patients with MDS.

## 4. Conclusion

Ph-positive MDS is a very rare disease. Ph may aid in the malignant progression of MDS leaving such patients with a very poor prognosis. TKIs plus chemotherapy followed by allogeneic hematopoietic stem cell transplantation has provided these patients with satisfactory outcome, which neither chemotherapy nor TKIs alone is able to do.

## Author contributions

Conception and design: Shasha Qi, Feiqing Wang, Yang Liu, Yanju Li.

Acquisition of data: Shasha Qi, Jiangyuan Zhao, Yan Wang, Songsong Huang, Wenxiu Yang, Yanling Li, Yong Shen, Yi Huang, Mei Zhao.

Analysis and interpretation of data: Shasha Qi, Feiqing Wang, Chike Zhang, Jianing Zhao, Xu Yang, Rui Gao, Ying Chen, Peng Zhao, Fengqi Zhang, Ping Wang, Yan Zhang, Hanbo Dou.

Drafting manuscript and review it: Shasha Qi, Feiqing Wang, Yang Liu, Jishi Wang, Yanju Li.

Final approval of the version to be submitted: Yang Liu, Jishi Wang, Yanju Li. All authors approved the final manuscript.

**Conceptualization:** Feiqing Wang, Shasha Qi, Yang Liu, Yanju Li.

**Data curation:** Chike Zhang, Feiqing Wang, Fengqi Zhang, Hanbo Dou, Jiangyuan Zhao, Jianing Zhao, Mei Zhao, Peng Zhao, Ping Wang, Rui Gao, Shasha Qi, Songsong Huang, Wenxiu Yang, Xu Yang, Yan Wang, Yan Zhang, Yanju Li, Yanling Li, Yi Huang, Ying Chen, Yong Shen.

**Formal analysis:** Feiqing Wang, Shasha Qi, Yanju Li.

**Funding acquisition:** Yanju Li.

**Investigation:** Shasha Qi, Yanju Li.

**Methodology:** Shasha Qi, Yanju Li.

**Resources:** Shasha Qi, Yanju Li.

**Writing – original draft:** Feiqing Wang, Jishi Wang, Shasha Qi, Yang Liu, Yanju Li.

**Writing – review &amp; editing:** Jishi Wang, Yang Liu, Yanju Li.
